# Sleep deprivation alters task‐related changes in functional connectivity of the frontal cortex: A near‐infrared spectroscopy study

**DOI:** 10.1002/brb3.2135

**Published:** 2021-06-22

**Authors:** Peter Mukli, Tamas Csipo, Agnes Lipecz, Orestis Stylianou, Frigyes Samuel Racz, Cameron D. Owens, Jonathan W. Perry, Stefano Tarantini, Farzaneh A. Sorond, Jeremy M. Kellawan, György Purebl, Yuan Yang, William E. Sonntag, Anna Csiszar, Zoltan I. Ungvari, Andriy Yabluchanskiy

**Affiliations:** ^1^ Oklahoma Center for Geroscience and Healthy Brain Aging Department of Biochemistry and Molecular Biology University of Oklahoma Health Sciences Center Oklahoma City OK USA; ^2^ Department of Physiology Faculty of Medicine Semmelweis University Budapest Hungary; ^3^ International Training Program in Geroscience Doctoral School of Basic and Translational Medicine/Department of Public Health Semmelweis University Budapest Hungary; ^4^ Division of Clinical Physiology Department of Cardiology Faculty of Medicine University of Debrecen Debrecen Hungary; ^5^ Department of Ophthalmology Josa Andras Hospital Nyiregyhaza Hungary; ^6^ Institute of Translational Medicine Semmelweis University Budapest Hungary; ^7^ Department of Health Promotion Sciences College of Public Health University of Oklahoma Health Sciences Center Oklahoma City OK USA; ^8^ Division of Stroke and Neurocritical Care Department of Neurology Northwestern University Feinberg School of Medicine Chicago IL USA; ^9^ Department of Health and Exercise Science University of Oklahoma Norman OK USA; ^10^ Institute of Behavioral Sciences Semmelweis University Budapest Hungary; ^11^ Stephenson School of Biomedical Engineering The University of Oklahoma Tulsa OK USA; ^12^ International Training Program in Geroscience Theoretical Medicine Doctoral School/Departments of Cell Biology and Molecular Medicine and Medical Physics and Informatics University of Szeged Szeged Hungary

**Keywords:** functional connectivity, near‐infrared, neuropsychological tests, sleep deprivation, spectroscopy

## Abstract

Sleep deprivation (SD) is known to be associated with decreased cognitive performance; however, the underlying mechanisms are poorly understood. As interactions between distinct brain regions depend on mental state, functional brain networks established by these connections typically show a reorganization during task. Hence, analysis of functional connectivity (FC) could reveal the task‐related change in the examined frontal brain networks. Our objective was to assess the impact of SD on static FC in the prefrontal and motor cortices and find whether changes in FC correlate with changes in neuropsychological scores. Healthy young male individuals (*n* = 10, 27.6 ± 3.7 years of age) participated in the study. A battery of tests from the Cambridge Neuropsychological Test Automated Battery (CANTAB) and 48 channel functional near‐infrared spectroscopy (fNIRS) measurements were performed before and after 24 hr of SD. Network metrics were obtained by graph theoretical analysis using the fNIRS records in resting state and during finger‐tapping sessions. During task, SD resulted in a significantly smaller decrease in the number and strength of functional connections (characterizing FC) in the frontal cortex. Changes in the global connection strengths correlated with decreased performance in the paired association learning test. These results indicate a global impact of SD on functional brain networks in the frontal lobes.

## Graphical abstract

To assess the impact of sleep deprivation on static functional connectivity in the frontal lobe, functional near‐infrared spectroscopy and graph theoretical analysis were utilized before and after 24 hr sleep deprivation in healthy young male participants. Deprivation of sleep resulted in a significantly smaller task‐related decrease in the number of functional connections that correlated with decreased performance in the paired association learning test. These results indicate the global impact of sleep deprivation on functional brain networks in the frontal lobes.

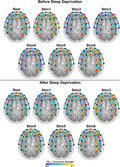

## 1 INTRODUCTION

Sleep is a vital homeostatic function (Tononi, 2012) for the central nervous system (CNS), and poor quality of sleep has been a growing health problem in Western countries (Chattu et al., 2018). Sleep deprivation (SD) has a high prevalence (32%–39%) among 18–65 years old adults (Watson et al., 2015) and has a significant impact on the public health and the economy (Grewal & Doghramji, 2017). Short sleep duration (<7 hr [Hirshkowitz et al., 2015]) is strongly associated with fatigue, impaired concentration, depression, and an overall decreased cognitive performance (Ben Simon et al., 2020; Krause et al., 2017; Nir et al., 2017). SD may contribute to the development of psychosis (Meyhöfer et al., 2017), and as recent studies suggest may have a role in the reduced clearance and increased accumulation of β‐amyloid, thus possibly contributing to a spectrum of changes from mood disturbances to the development of Alzheimer disease (Shokri‐Kojori et al., 2018). Besides the direct effect on the CNS, abundant studies demonstrate that SD is also associated with an increased risk of obesity, type 2 diabetes mellitus, and other cardiovascular conditions through, possibly, a profound effect of SD on metabolic processes (Reutrakul & Van Cauter, 2018). The deleterious effect of SD on general health further alters normal CNS functioning. In turn, impaired CNS functioning negatively affects general health, which leads to sleep disturbances (Muscogiuri et al., 2019) and therefore increases the risk of SD. Thus, these interactions create a vicious circle, which may escalate the negative consequences of SD. Despite its prevalence and clinical importance, the mechanisms underlying sleep deprivation‐induced decline in cognitive performance are poorly understood.

Several functional neuroimaging studies have reported global and local decreases of neural activity in the state of sleep deprivation (Boonstra et al., 2007). These methods used in these studies also allow for assessment of functional connectivity (FC) of the brain describing the dynamic connections between brain regions with the aid of measures capturing the statistical relationship between the simultaneously recorded neurophysiological processes (Biswal et al., 1995). As far as macroscopic scales concerned, sleep deprivation‐induced deficits in attention and executive function imply the specific involvement of the prefrontal cortex (PFC) (Thomas et al., 2000). These cognitive processes require the cooperation between brain regions that give rise to specific networks dedicated to that mental function (Bullmore & Sporns, 2009). Based on the statistical relationship between signals obtained from different brain regions acquired by functional magnetic resonance imaging (fMRI), altered FC has been reported between the corresponding resting‐state networks (Javaheripour et al., 2019). Specifically, the default mode network (DMN, a functionally connected neural network prevailing in the resting state (Greicius et al., 2003)), which is important for normal mental function (Andrews‐Hanna, 2012), was found susceptible to sleep deprivation (Chen et al., 2018).

In order to better understand the mechanisms contributing to sleep deprivation‐induced changes in functional connections and their link to impaired cognitive performance in real‐life situations (e.g., decision making), additional studies are needed. Importantly, studying task modulations of brain connectivity is essential. Changes in FC can be detected using functional near‐infrared spectroscopy (fNIRS). The advantage of fNIRS lies in its ability to directly capture local changes in cerebral hemodynamics by continuously monitoring concentrations of oxygenated and deoxygenated hemoglobin in the cerebral cortex (Bunce et al., 2006; Csipo, Lipecz, et al., 2019; Csipo, Mukli, et al., 2019; Mukli et al., 2018). fNIRS is a cost‐effective and portable alternative to fMRI (Villringer & Chance, 1997) and has higher temporal resolution too (Quaresima & Ferrari, 2019). The fMRI approach—considered currently as the gold‐standard in neuroimaging—has several practical disadvantages: It imposes restrictions due to the prolonged procedural duration and limits the types of neuronal stimulation to those that can be performed in an essentially immobilized subject. In contrast, fNIRS is ideal for longitudinal studies and is much less demanding on the study subjects. Although fNIRS is an excellent tool to investigate resting‐state and task‐related functional connectivity either in the whole brain cortex (Mesquita et al., 2010) or only in the (pre)frontal cortex (Racz et al., 2017), the impact of sleep deprivation on spatial patterns of cerebral hemodynamics measured by fNIRS is undocumented in the literature.

The aim of this study was to assess the effect of sleep deprivation on the local and global properties of the functional brain networks of the frontal lobe using fNIRS, both in the resting state and during motor task. To test the hypothesis that after sleep deprivation, task‐related changes of static FC are different compared to the nonsleep‐deprived state, we examined the FC by applying a finger‐tapping paradigm that is considered to activate specific regions of the motor cortex. The functional network of the frontal lobe was reconstructed using cerebral hemodynamic signals recorded from the corresponding brain region. These localizations determined the node of the graph representing the functional brain network of interest, while the strength of relationship—estimated by Pearson's correlation—between each pair of signals defined an edge weight in the graph. Finally, we examined the relationship between parameters of FC and several domains of cognitive function to determine whether SD‐related changes in neuropsychological test scores are associated with altered brain network properties.

## 2 MATERIALS AND METHODS

### 2.1 Study participants and design

Ten young healthy adults (males, age of 27.6 ± 3.7 years, one left‐handed participant) were recruited for this study. None of the volunteers reported smoking, drug abuse, excessive alcohol consumption, and relevant medical history including neurological, psychiatric diseases, or any other significant condition (cardiovascular disease, diabetes, cancer, infection within two weeks of the examination date). All participants had a college degree. The study protocol was approved by the Institutional Review Board of the University of Oklahoma Health Sciences Center, and all participants provided a written informed consent prior to participation in the study.

Subjects were asked to record their sleeping pattern using a personal diary log for 7 days prior to sleep deprivation. The sleep quality was characterized by self‐judgment in terms of approximate time of falling asleep, the time it took to fall asleep, number of awakenings at night, the time of final wake‐up in the morning, any condition during these days that could affect sleep (including illness, emotional stress, disturbances, etc.), amount of consumed alcoholic and caffeinated beverages, and average sleepiness on a scale 1–10. All participating subjects were requested to maintain a sleep duration of no less than 7 uninterrupted hours of sleep daily over the course of 7 days. Baseline measures were acquired from all subjects 3–5 days before the scheduled date of SD. On the day of sleep deprivation, subjects arrived at the testing facility where they stayed awake until the following morning, when the final assessments were performed. Subjects were asked not to consume any substances affecting cognitive performance (caffeinated beverages) for at least 24 hr prior to measurements. We measured blood pressure before and after SD, and other basal physiological parameters obtained in this cohort have been reported (Csipo et al., 2021). Adherence to the protocol was ensured by an investigator who was present in the testing facility overnight.

### 2.2 fNIRS: setup and protocol

In this study, functional NIRS measurements were performed using a NIRScout platform (NIRx Medical Technologies LLC). The system was equipped with 16 sources (F3, AF7, AF3, Fz, Fpz, AF4, F4, AF8, FC6, C4, FC2, CP2, FC1, CP1, C3, FC5) emitting light at two different wavelengths (760 and 850 nm) and 16 photodetectors (F5, F1, Fp1, AFz, F2, Fp2, F6, AFF6h, C6, CC4, CP4, C2, C1, FC3, CP3, C5) defining 48 channels. A 128‐port Easycap (Easycap GmbH) was mounted over the head covering the area of the international 10–10 system. Optode localization was guaranteed by aligning the line between Fpz and Iz ports on the headcap with the midsagittal plane and aligning the optode in the Fpz position of the cap with Fpz on the participant's head. Average source–detector distance was 3 cm, and the variability of the distance between optodes was limited by setting up the cap with custom‐made spacers, provided by the manufacturer. This setup of optodes was capable of probing the PFC, dorsolateral PFC, and also the medial motor cortex which was confirmed by the projection of channel position to the cortical surface within the Montreal Neurological Institute coordinate space (Supplemental Table 1 in Csipo et al., 2021) (Aasted et al., 2015; Tsuzuki et al., 2012). The optical density changes of the probed tissue were determined by the measured light intensity values detected at a sampling frequency of 3.9 Hz.

**FIGURE 1:**
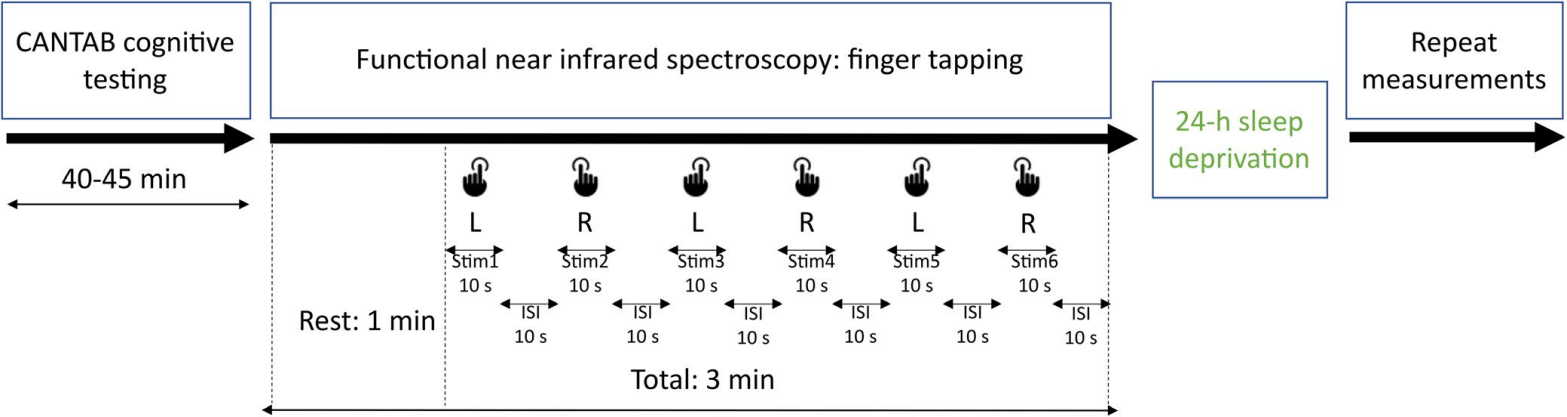
*Illustration of measurement protocol and finger‐tapping task*. After giving informed consent, participants completed CANTAB testing that was followed by functional near‐infrared spectroscopy (fNIRS) measurements. fNIRS signals were recorded during one‐minute resting state (baseline) and during 10 s length stimuli for left and right hand alternatively. Subjects tapped their index finger against the surface according to auditory commands which indicated the beginning (“left” or “right”) and the end of the task (“stop”). Stimuli were repeated three times for each hand denoted by Stim1, Stim2, …Stim6. The interstimulus interval (ISI) was 10 s. All measurements were repeated after 24 hr sleep deprivation

The fNIRS recordings (available on PhysioNet) were acquired in a quiet and darkened room, while the subjects were seated in a comfortable armchair. Changes in cerebral hemodynamics were captured during a one‐minute resting state followed by a finger‐tapping task (Figure 1). In brief, each subject was presented with an auditory command to perform three sets of 10 s using the left index finger (Stim1, Stim3, and Stim5) and three sets of 10 s using the right (Stim2, Stim4 and Stim6) index finger‐tapping task with 10 s interstimulus periods between each task.

### 2.3 Data preprocessing

The measured fNIRS signal comprises not only components associated with functional brain activation but also systemic physiological processes with characteristic frequencies. The majority of these oscillations originate from heart pulsation, breathing, and autonomic nervous system functions which contribute to scalp hemodynamics (Scholkmann et al., 2014). Artifacts were removed (a) by thresholding after discrete wavelet transformation (Molavi & Dumont, 2012) and then (b) in the frequency domain using a 5th order Butterworth filter with a low‐pass frequency of 0.4 Hz (Tian et al., 2009). Preprocessed optical densities were converted into concentration changes of oxy‐ (HbO) and deoxyhemoglobin (HbR) according to the modified Beer–Lambert law (Cope et al., 1988; Kocsis et al., 2006), and total hemoglobin (HbT) was calculated as the sum of HbO and HbR. For functional connectivity analyses, we applied correlation‐based signal improvement (CBSI) to enhance the representation of signal components associated with task‐related brain activity (Cui et al., 2010). This method was originally developed for the finger‐tapping paradigm, and it is based on the principle that fluctuations of HbO and HbR become more anticorrelated during evoked hemodynamic responses due to the nature of the neurovascular coupling (NVC) (Cui et al., 2010).

### 2.4 Graph theoretical analysis

We determined the functional brain networks using preprocessed HbT time series, as described in Ref. (Racz et al., 2017). We used the entire signal from the stimulus epochs and chose a segment with the same length (10 s) from the middle of the resting‐state period. The strength of functional connection between brain regions (channels) was characterized in terms of temporal correlation of the measured signal pairs similarly to previous fNIRS studies (Mesquita et al., 2010; Novi et al., 2016). For each subject and each state, we obtained the connection matrices of Pearson's correlation coefficients representing all pairwise combinations of channels. In order to eliminate spurious connections, we replaced all negative and certain positive coefficients with 0 according to three different, complementary thresholding schemes that were applied separately (van den Heuvel et al., 2017). In case of absolute thresholding, coefficients less than a predefined positive value (between 0.15 and 0.75 with a step of 0.05) were discarded from further calculations. During cost thresholding, a certain arbitrarily defined fraction of connections was preserved reflecting the strongest relationships (between 10% and 85% with a step of 5%) yielding a connection matrix with the highest value of Pearson's coefficients. The third approach used a surrogate thresholding scheme by eliminating the statistically nonsignificant links based on the *p*‐value of individual correlations (α_s_ = 0.05). Since the obtained matrices are symmetric, all approaches allow for reconstructing undirected graphs, reflecting the linear statistical interdependence between the examined brain regions.

In the next step, we defined the graph theoretical parameters both for binary and weighted undirected networks (Rubinov & Sporns, 2010). During binarization, all nonzero elements of the thresholded connection matrix were replaced by 1, while the latter representation considered these coefficient values as edge weights in the graph. Functional brain networks were characterized using common metrics: density (*D*), the clustering coefficient (*C*), and the global efficiency (*E*). These parameters can be calculated for each node (local, denoted by a subscript loc) and for the whole network as an average across the nodes; the weighted parameter is denoted by a left superscript W.

Local weighted density is a fraction of connection strength to the maximal connection strength for each node and it is the average of the corresponding edge weights. For node *i*, it is calculated as

(1) $${}^{W}D_{\mathrm{loc}}(i)=\frac{1}{N‐1}\sum_{j\in N‐1}c_{\mathrm{ij}},$$

where *N* is the total number of nodes, *c_ij_* is the connection strength between nodes *i* and *j* that could take any value for weighted networks between 0 and 1. Conversely, for binary networks, *c_ij_* is equal to 1 if a link exists between nodes *i* and *j*, and 0 otherwise, we call the quantity $\sum_{j\in N‐1}c_{\mathrm{ij}}$ as the node degree representing the number of connections. The global density (weighted networks) or average node degree (binary networks) is the simple functional connectivity parameter:

(2) $$D={\bar{D}}_{\mathrm{loc}}=\frac{1}{N}\sum_{i\in N}D_{\mathrm{loc}}(i)$$

that represents the overall “wiring cost” of the examined network.

We also computed the binary global clustering coefficient, which expresses the average probability that a link exists between two nodes if there is another node in the graph, which is connected to both. In other words, it is equal to the average of local clustering coefficient according to

(3) $$C={\bar{C}}_{\mathrm{loc}}=\frac{1}{N}\sum_{i\in N}\frac{1}{k_{i}(k_{i}‐1)}\sum_{j,h\in N}c_{\mathrm{ij}}c_{\mathrm{ih}}c_{\mathrm{jh}},$$

where *k_i_* is the degree of node *i*, hence *C* reflects how the neighboring nodes in the network form connected groups. Therefore, *C* is a measure of functional segregation for the whole network.

By definition, the architecture of a functional network is more efficient if the average shortest path length between all pairs of nodes is smaller (Watts & Strogatz, 1998). The inverse of this parameter yields a measure that is referred to as global efficiency (Latora & Marchiori, 2001) defined as

(4) $$E=\frac{1}{N}\sum_{i\in N}\sum_{j\in N,i\neq j}\frac{d_{\mathrm{ij}}^{‐1}}{n‐1},$$

where *d_ij_* is the shortest path between nodes *i* and *j* of a binary network. Efficiency captures functional integration by describing the effectiveness of information transfer between distinct nodes of the given network.

### 2.5 Cognitive assessment

We assessed cognitive performance with the aid of Cambridge Neuropsychological Test Automated Battery (CANTAB). To reveal any sensorimotor deficits or lack of comprehension, the testing began with the Motor Screening Task (MOT). After successful completion of MOT, the following domains of cognitive functions were examined in this study: attention and psychomotor speed (Rapid Visual Processing, RVP test and Reaction Time, RTI tests), spatial working memory (SWM test), visual memory, short‐term recognition, and visual information matching (Paired Associates Learning, PAL and Delayed Matching to visual Sample, DMS tests). All participants performed tests uninterrupted using the touchscreen 10.5” iOS tablet device running the CANTAB application.

### 2.6 Statistical analyses

Data preprocessing and analyses were carried out using custom scripts written by the authors in Matlab (Mathworks, Inc.). Functions for calculating the graph theoretical parameters were obtained from the Brain Connectivity Toolbox (Rubinov & Sporns, 2010). Since the distribution of these variables was not normally distributed in most of the cases, normality (Shapiro–Wilk) and sphericity (Mauchley) were also checked after logarithmic transformation with α_s_ = 0.05 (significance level). The effect of sleep state and stimulus was assessed with the aid of a two‐way repeated‐measures analysis of variance (ANOVA) where sleep state had two levels (before SD, after SD) and task state had seven levels (*Rest*, *Stim1*‐*Stim6*). Difference of means was considered significant in case of *p* < .05 confirmed by using Dunnett's post hoc test which used values obtained in resting state as controls to the compare with task states. All statistical tests were performed with TIBCO Statistica 13.4.

## 3 RESULTS

### 3.1 Study participants

Features of sleep quality and wakefulness based on the 7‐day diary log and baseline physiological parameters are summarized in Table 1. While all participant sufficiently adhered to the study protocol, one subject was excluded due to data poor quality defined by high variability (coefficient of variation >7.5% [Zimeo Morais et al., 2018]) in more than 80% of channels. There was no statistically significant difference in the measured arterial blood pressure before and after sleep deprivation.

**TABLE 1:** Quality of sleep–wake cycle and baseline physiological parameters of the subjects

Duration of sleep	7.9 ± 0.6 hr
Time needed to fall asleep	11.3 ± 9.6 min
Wake up during night	0.7 ± 0.6 times per night
Daily stress	0.04 ± 0.07 times per day
Daily coffee or alcoholic beverages	0.6 ± 0.6 daily
Sleepiness on a scale from 1 (nonsleepy) to 10 (very sleepy)	3.1 ± 0.9
Blood pressure before sleep deprivation	
Systolic	119 ± 6 mmHg
Diastolic	80 ± 6 mmHg
Blood pressure after sleep deprivation	
Systolic	116 ± 8 mmHg
Diastolic	71 ± 12 mmHg

### 3.2 Global connectivity measures

**FIGURE 2:**
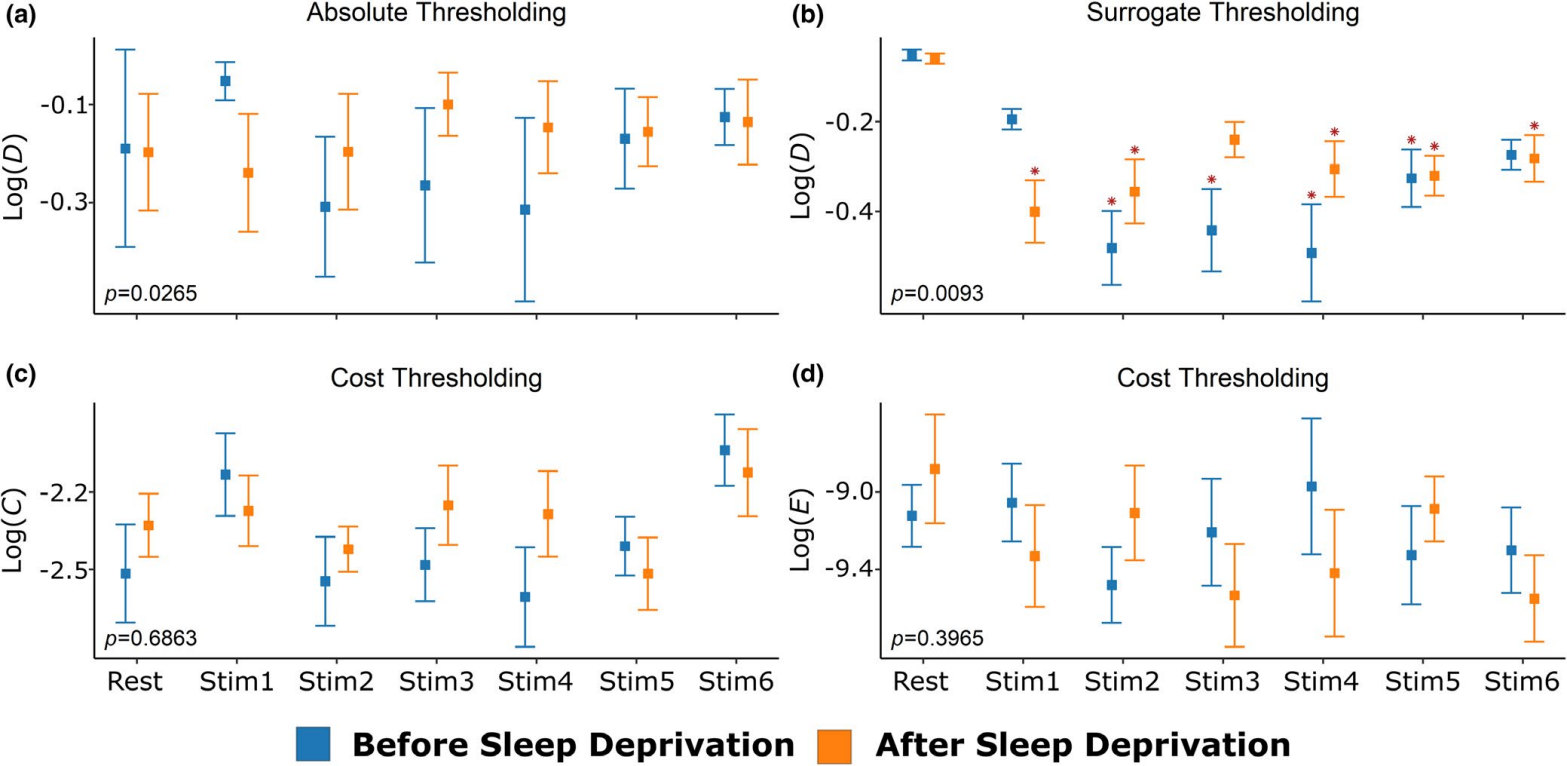
*Global parameters of functional connectivity*. (a) Changes of average node degree, *D*, associated with stimulus and sleep deprivation are depicted for absolute thresholded networks. (b) The same effect is shown for *D* in the case of surrogate thresholded networks. (c) Global clustering coefficient, *C*, for cost‐thresholded networks. (d) Global efficiency, *E*, for cost‐thresholded networks. Vertical bars denote standard deviation; orange: before SD, blue: after SD. For all panels, network metrics are shown after logarithmic transformation. Groups were compared by repeated‐measures analysis of variance (RM‐ANOVA, *p*‐values are displayed in the lower left corner), and significant differences were identified by Dunnett's post hoc test considering the corresponding resting period—of the same sleep state—as control. Asterisks on panel B indicate Dunnett's *p* < .05, while there were no significant differences with other thresholding scheme. For further details see text

Effect of finger tapping compared to a resting state before and after SD is presented in Figure 2. First, different task and sleep states were compared in terms of the average node degree (global density calculated on binary networks). In spite of *p* = .027 referring to the significant interaction of these effects (Figure 2a) in the case of absolute thresholded networks, Dunnett's post hoc tests did not confirm any differences. Moreover, neither task nor sleep were found as significant on their own, as main effects. These results suggest that the functional connectivity evolves according to a characteristically different but not consistent pattern across different task states after sleep deprivation compared to the nonsleep‐deprived state. It is of note that threshold had a marked effect on *D*; thus, it was included as a third repeated‐measures factor in the general linear model.

Contrarily, when we examined surrogate thresholded networks, a marked decrease in connectivity (Figure 2b) was found. Finger tapping was associated with a reduced number of connections (*p* = .00001), and Dunnett's test confirmed significant differences. Two‐way repeated‐measures ANOVA also revealed a significant effect of the interaction between sleep state and stimulus levels on *D* for most of the post hoc rest versus task comparisons. Since spurious connections were omitted from characterizing surrogate thresholded networks, task state was characterized by less significant links between the monitored brain regions. Apparently, task‐related changes of *D* were smaller after SD in comparison with the preceding state. Comparing the average of weighted density (connection strength) also revealed the statistically significant effect of the interaction between sleep and task (*p* = .012).

In order to evaluate functional segregation and functional integration of different networks, it was necessary to apply cost thresholding. Thus, the same number of functional connections enabled a comparison of the global clustering coefficient and global efficiency. Although these results also depend on the chosen cost value, the global parameters of functional connectivity obtained for cost‐thresholded networks are calculated and shown after summing these parameters across networks determined at different cost values between 10% and 85% similarly to Racz et al. (2020). Statistical analysis did not reveal any effect neither of sleep deprivation nor of task, not even as a combined effect. This reflects the functional stability of the investigated frontal network at considerably different wiring cost values. These results justified the usage of parameters derived from *D* in subsequent analyses.

**FIGURE 3:**
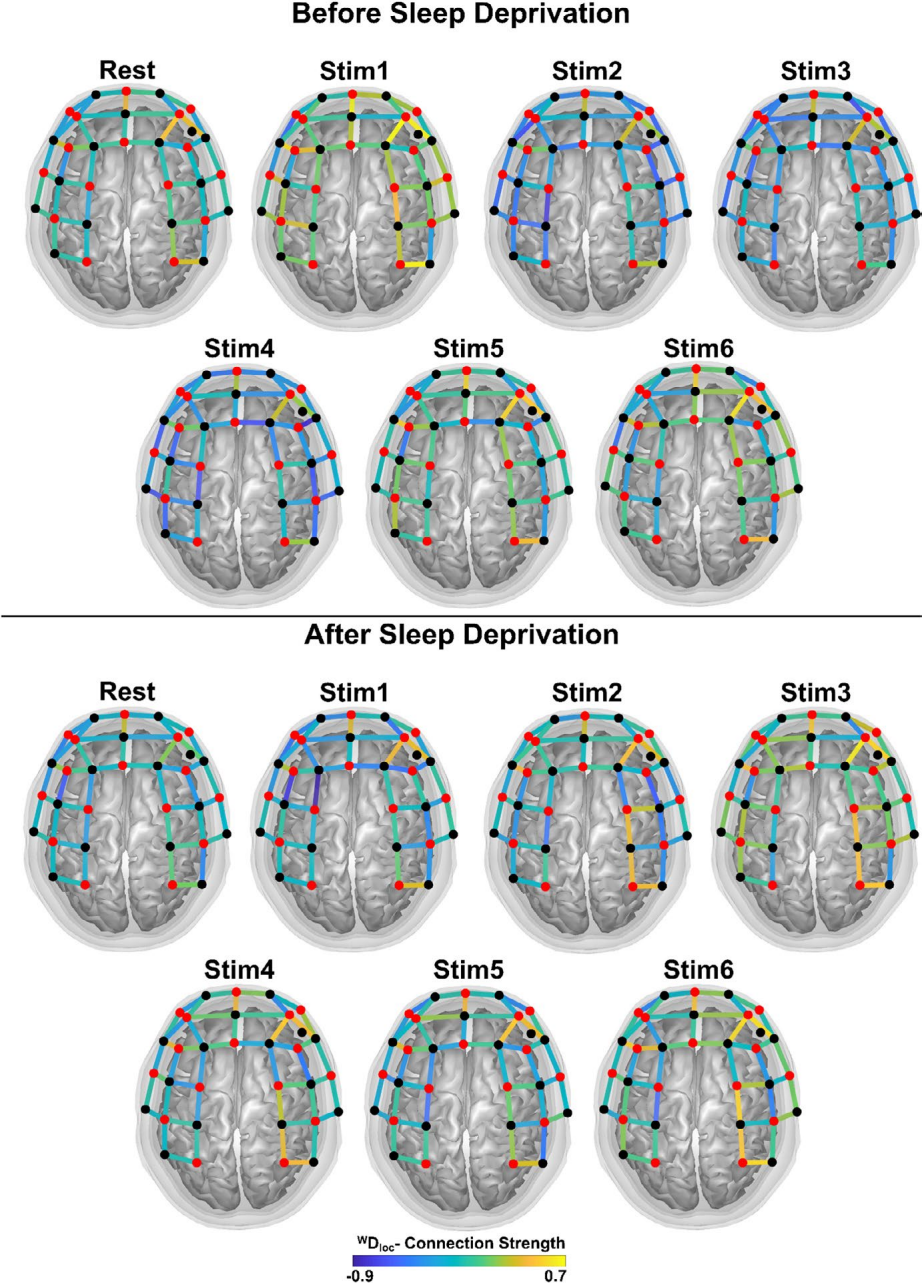
*Connection strength during rest and finger tapping, before and after sleep deprivation*. Position of optodes covering prefrontal and motor cortices is shown together with sources (red dots) and detectors (black dots) defining nodes of the examined network. Local weighted densities averaged across all subjects for each channel are mapped—after logarithmic transformation—as the color of edges representing connection strength of fNIRS channels. The standard channel positions in the current montage and the relation of actual channel layout to the brain were visualized by AnalyzIR toolbox (Santosa, 2018)

**FIGURE 4:**
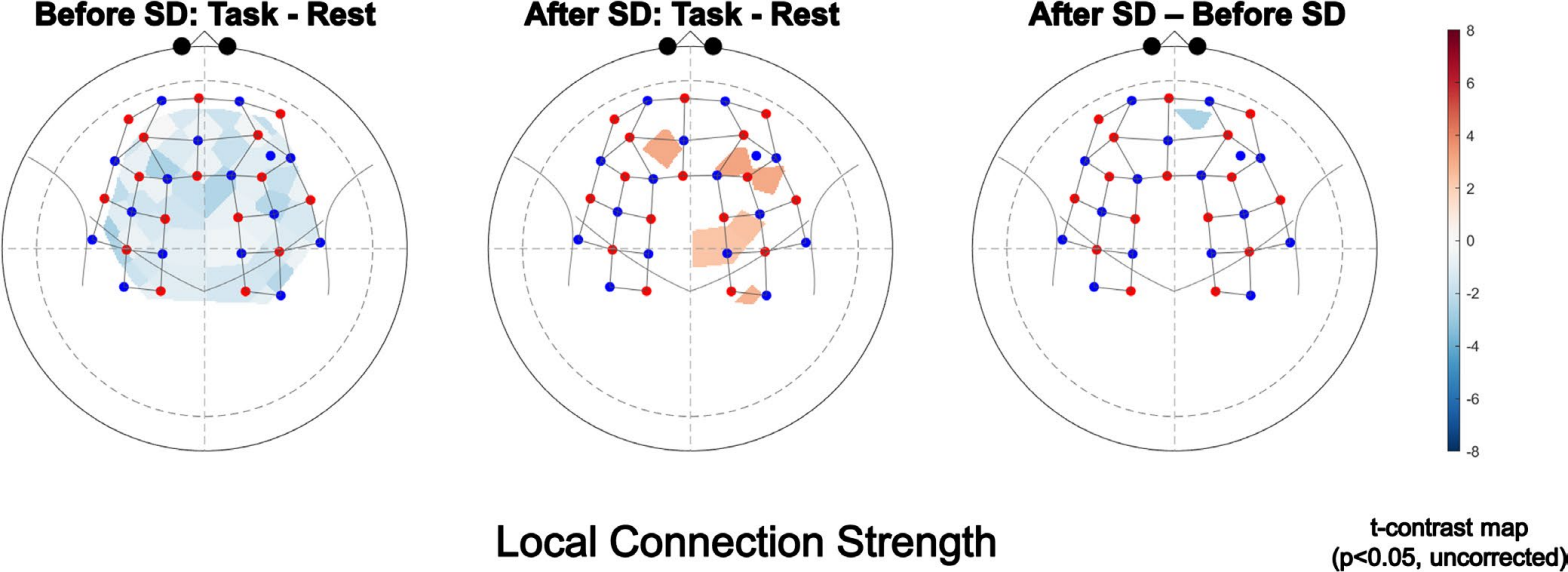
*Effect of localization for connection strength*. T‐contrast maps show the effect of task before (left) and after (middle) sleep deprivation (SD), and the effect of SD (right) on local connection strength. Resting state was subtracted from the average of all stimulation periods and was tested whether it significantly differs from 0 (left and middle panel) or to each other (pairwise comparison, right panel). The threshold for significance was set to *p* < .05 (uncorrected). Please note the mild global decrease in FC before SD (blue shades) and the increased connectivity limited to several regions on the right hemisphere (shades of red). After correcting for multiple comparisons (by controlling false discovery rate), none of the effects were significant. The standard channel positions in the current montage and the relation of actual channel layout to the brain were visualized by AnalyzIR toolbox (Santosa, 2018)

We evaluated the effect of task and sleep state on functional connectivity also at the local level by comparing connection strengths in surrogate thresholded networks; the overall changes of *^W^D_loc_* are depicted in Figure 3. Statistical evaluation of task and sleep deprivation effect are shown on Figure 4. In these calculations, weighted networks were used that allowed for assessment of nodal connection strength at a finer resolution compared to node degree (obtained after binarization). In the nonsleep‐deprived state, connection strength increased transiently (Stim1), followed by a decrease during repeated finger tapping (Stim2‐Stim4) and an increase at the end (Stim5‐Stim6) for the majority of channels. Conversely, after sleep deprivation, the elevated connection strengths in the resting state were rather preserved and even increased during the finger‐tapping sessions. Channels in the motor cortex exhibited increased *^W^D_loc_* in particular, albeit none of such channel‐wise effects reached the level of significance due to the moderate between‐subjects concordance (Kendall's W = 0.3186). In spite of these noticeable patterns of local functional connectivity changes, none of these effects could be localized.

### 3.3 Relationship between cognitive performance and functional connectivity

**FIGURE 5:**
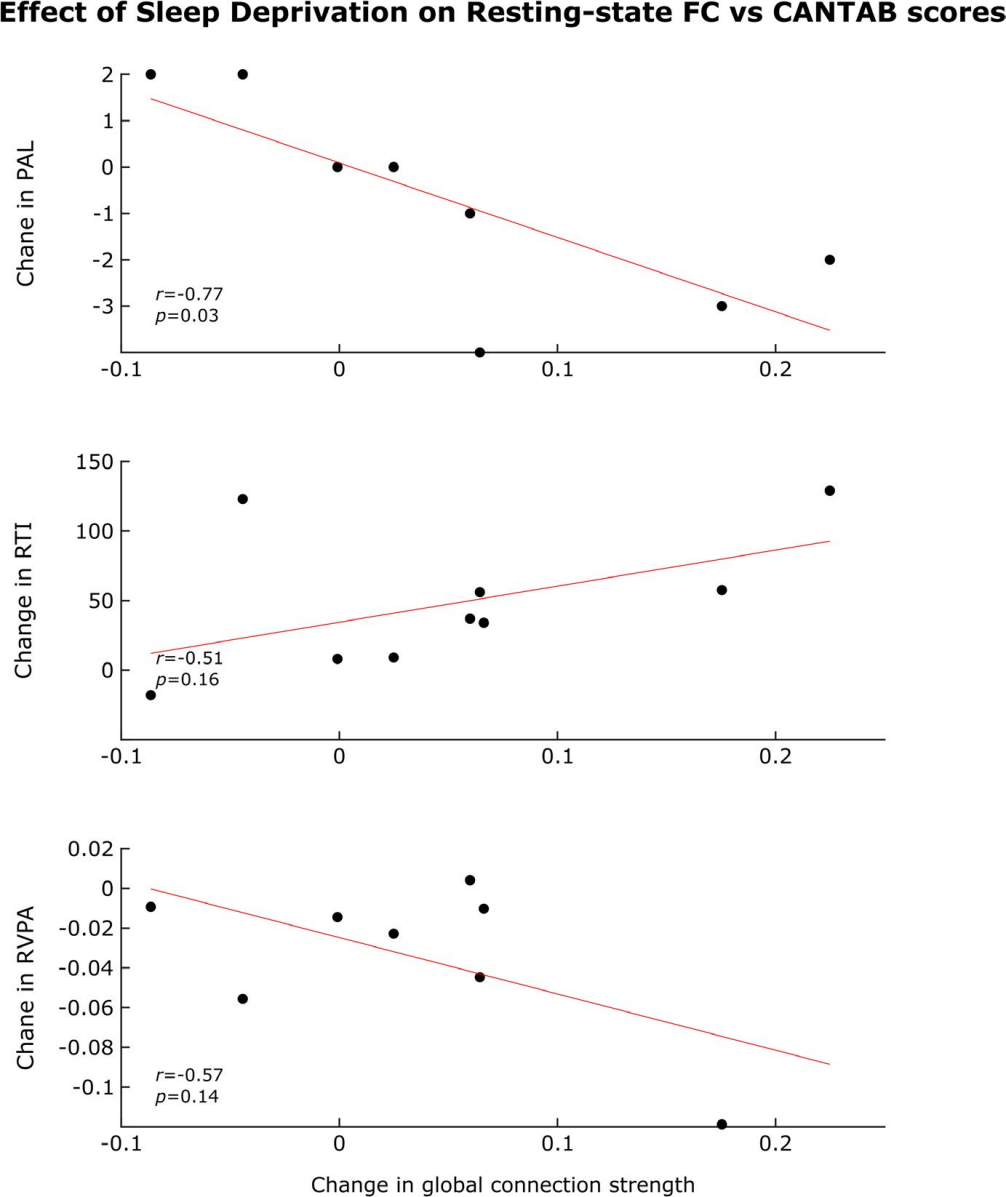
*Correlation between global connectivity metrics and cognitive scores*. The variable used to describe the impact of sleep deprivation on resting‐state brain networks was change in global connection strength calculated from surrogate thresholded networks. *x*‐axis: $\Delta^{W}D={}^{W}{\bar{D}}_{\mathrm{after}}‐{}^{W}{\bar{D}}_{\mathrm{before}}$, where ${}^{W}{\bar{D}}_{\mathrm{before}}$ refers to the difference between average task state and resting state before SD and ${}^{W}{\bar{D}}_{\mathrm{after}}$ is defined similarly after SD; *y*‐axis: the change in Paired Association Learning—First Attempt Memory (PAL) score (panel A), the change in Reaction Time (RTI) score (panel B) and the change in Rapid Visual Processing and Attention (RVPA) score (panel C). Black dots represent corresponding brain network metrics—neuropsychological score for each individual; in case of one subject, we could not obtain a valid PAL score and a valid RVPA score. The displayed *p*‐values are uncorrected

To evaluate the association between the difference (∆) in ^W^ *D* and in neuropsychological scores obtained by CANTAB tests (Figure 5), we defined a change in global connection strength by subtracting the resting‐state *^W^D_r_* values from the *^W^D_t_* averaged across the corresponding task states. This calculation was done both before and after sleep deprivation yielding ${}^{W}{\bar{D}}_{\mathrm{before}}$ and ${}^{W}{\bar{D}}_{\mathrm{after}}$; and ∆^W^ *D* was defined as a difference between the values obtained this way: $\Delta^{W}D={}^{W}{\bar{D}}_{\mathrm{after}}‐{}^{W}{\bar{D}}_{\mathrm{before}}$. Thus, we obtained a measure representing the impact of SD on the global network response of the probed brain regions. A significant relationship was revealed only with a change in PAL (Paired Association Learning) First Attempt Memory score, a measure of executive function. A clear difference between task‐related change of connection strength before and after SD was observed in participants whose second PAL score showed a decrease compared to that achieved during the first visit. Since the low number of subjects did not permit assessing brain–behavior correlation at a local level, we only evaluated the influence of global network metrics on cognitive performance.

## 4 DISCUSSION

In this study, we tested the novel hypothesis that deprivation of sleep does affect functional brain networks in the frontal lobe captured by fNIRS. We confirmed that sleep deprivation significantly alters task‐related changes in functional connectivity. In essence, the observed global network response in the sleep‐deprived state is featured by significantly less decrease in the average number of functional connections. We also found a significant negative correlation between changes in PAL score and task‐related alteration of global connection strength.

Brain networks of the frontal lobe were reconstructed using temporal correlation of simultaneously recorded fNIRS signal, a broadly utilized approach to investigate functional organization underlying mental processes (Biswal et al., 1995; Friston et al., 1993). Hemoglobin signals were obtained after reducing the contribution of systemic influences and eliminating artifacts (Cooper et al., 2012). For each brain state and each pair of NIRS channels, ten‐second length of preprocessed HbT time series was included in the graph theoretical analysis. This time period was used in previous fNIRS studies utilizing finger‐tapping paradigm and was sufficient to capture the NVC‐related hemodynamic fluctuations (Kashou et al., 2016), particularly after signal conditioning with CBSI (whose effectiveness was shown previously [Racz et al., 2017]). Due to differences in stimulus sessions (left vs. right), we did not apply block averaging at the level of channels (neither fNIRS signals nor connection matrices).

The chosen parameter to describe the strength of the relationship between brain regions was Pearson's correlation coefficient which is common in fNIRS studies of FC (Li & Qiu, 2014; Niu & He, 2014; Niu et al., 2012; Novi et al., 2016). Due to differences in stimulus sessions (left vs. right), we did not apply block averaging at the level of channels (neither fNIRS signals nor connection matrices). Graph theoretical analyses were performed after determining the fraction of correlation coefficients defining a real functional link according to three different thresholding criteria. In the case of absolute thresholding, the investigated network became more connected—albeit not significantly—due to a widespread increase in the hemodynamic fluctuations as indicated by an elevated *D*. For example, a parallel increase in HbT in different brain regions is indicated by an increased correlation that can be due to simultaneously elicited neurovascular coupling responses similar to what Racz and colleagues found (Racz et al., 2017). In contrast, surrogate thresholded networks showed a task‐related decrease in average node degree which can be rather explained by an unrelated change between many brain regions with more correlated cerebral hemodynamics. The observed alterations in brain network topology suggest a parallel change of hemoglobin signals in many NIRS channels, albeit the underlying temporal dynamics show regional differences that could explain the nonsignificant correlations. This is reasonable since certain functional links between two probed brain regions may not be captured in case of a remarkable time delay affecting their correlation. These results could be attributed to the transient elimination of functional connections during finger‐tapping sessions that are part of resting‐state networks but not relevant to this experimental paradigm. Importantly, this interpretation is supported by the high activity of the brain in the resting state (Raichle & Mintun, 2006) exhibiting a dense network of functional connections, while the external stimulus elicits a dissociation between specific cerebral regions (Fox et al., 2005). Finally, analyses of cost‐thresholded networks did not reveal significant differences neither in clustering coefficient nor in efficiency. The inference is that any differences observed in absolute or surrogate thresholded network in terms of *C* or *E* might be attributed to a different number of connections in the compared network (van den Heuvel et al., 2017), not to the alteration of functional segregation or functional integration. The combination of different thresholding sheds light on the reason for heterogenous results obtained by previous studies and enables an enhanced interpretation. It is of note that statistical analyses were performed on data used for demonstrating local connectivity and correlation with cognitive performance changes were derived from weighted networks. While the latter approach preserves more information due to a finer resolution of connection strengths, binary networks provide more contrast for statistical comparisons (even weighted densities, that is, connection strength the interaction between sleep state and task is significant).

We observed heterogeneous spatial distribution regarding changes in local connectivity metrics across different brain states (Figure 2). To unravel the effect of SD on local connectivity, results were compared either in case of channels expected to be activated by a simple motor task or channels showing the largest functional hyperemia based on the same data. On the one hand, the finger‐tapping paradigm is expected to elicit hemodynamic responses, particularly in the contralateral motor cortex (Matsuzaki et al., 2010; Rogers et al., 2004), even though our results do not substantiate task‐related increase of connection strengths despite the largest response of the corresponding *^W^D_loc_* values. On the other hand, Csipo and colleagues reported attenuated neurovascular coupling responses in the ventromedial PFC after SD (Csipo et al., 2021). While this hemodynamic impairment might have an impact on the reorganization of functional connections, we found only nonsignificant effect of sleep and/or task (reflected by *p*‐values after correcting for multiple comparisons) in these affected brain regions. Though we used CBSI‐treated hemoglobin signals for FC calculations (instead of prewhitened detrended HbO signal from which NVC responses were derived in Csipo et al., 2021), the differences between localization of altered FC and decreased NVC responses could be attributed to the distinguished effect of SD on them. A plausible explanation is that attenuated NVC responses observed in part of the PFC contribute to local functional connectivity properties in all regions of the brain connected functionally to PFC. Alternatively, the depressed neuronal function might translate into a similar pattern of topological alterations, but the applied methods did not allow for direct assessment of such an effect. Nevertheless, our results indicate that global and local network properties seem to capture a rather widespread response of the probed frontal lobe regions beyond the ventromedial PFC. Although NIRS is an excellent tool to investigate resting‐state and task‐related FC either in the whole brain (Mesquita et al., 2010) or only in the (pre)frontal cortex (Racz et al., 2017), the impact of SD on spatial patterns of cerebral hemodynamics remains to be elucidated. However, there is a notable similarity with resting‐state fMRI measurements also demonstrating altered connectivity in the DMN (Chen et al., 2018).

SD significantly impaired reaction time (RTI) and sustained attention (RVPA), but it did not significantly affect other cognitive domains examined by the CANTAB test battery (data shown in Csipo et al., 2021). These results implicate attention deficit and decreased arousal being in agreement with current knowledge on the behavioral effects of SD (Alhola & Polo‐Kantola, 2007; Boonstra et al., 2007; Chua et al., 2014; Goel et al., 2009). Previous studies demonstrated that sleep is involved in the acquisition, maintenance, and retrieval of memories (Rauchs et al., 2005) as well as memory consolidation (Saxvig et al., 2008) and that sleep deprivation affects both working memory and long‐term memory processes. In this paper, we extended these investigations by examining the association of sleep‐deprived state with altered functional connectivity. Correlation analysis revealed a significant relationship between SD‐related change in *^W^D* and difference in RVPA score but only in the resting state (data not shown). The increase in task‐related changes in global connection strength showed a significant anticorrelation with PAL score (Figure 3) differences of the sleep‐deprived state compared to control assessment. In fact, this cognitive test can detect deficits in visual memory and new learning associated with subcortical brain structures as well. In line with this result, it can be speculated that the observed effect of prolonged wakefulness on task‐related changes of FC might be an indirect sign of altered cooperation with brain regions not examined in this study (due to the depth limitations of fNIRS), such as the hippocampus. The existing evidence support the theory that SD‐induced changes in the default mode network are likely causally linked to cognitive impairment (Chen et al., 2018).

Reduced activity in the frontal lobe was reported after sleep deprivation, which might be attributed to impaired endothelial function (Yeung et al., 2018). This interpretation is supported by attenuated NVC found in the PFC of our participants (Csipo et al., 2021) and previous study evidencing the acute endothelial dysfunction following SD (Calvin et al., 2014; Sauvet et al., 2010). We observed a smaller task‐related decrease in global connection strength due to preserved higher local connectivity. It suggests a redistribution of functional connections spreading across several sleep‐deprived brain regions. Actually, it may be shaped by the increased resting cerebral blood flow, also being considered as a consequence of prolonged wakefulness (Elvsashagen et al., 2019). Regarding recent studies investigating frontal brain networks, decreased connectivity in the PFC was found based on a NIRS study (Bu et al., 2017) and an electroencephalographic (EEG) study (Verweij et al., 2014). These results are not directly comparable since none of them utilized Pearson's correlation to describe functional connections.

This study demonstrated altered task‐related changes in functional connectivity suggesting acute dysregulation of physiological processes in the context of sleep deprivation. Considering further pathophysiological mechanisms related to sleep loss, disturbed sleep–wake cycles promote characteristic changes in the autonomic nervous system (Tobaldini et al., 2017). Falling asleep is accompanied by an increased parasympathetic tone that was likely absent in case of our participants (measured blood pressure changes were not significant). In fact, poor sleep quality and impaired autonomic control signaled by decreased heart rate variability have been associated with the increased risk of cardiovascular disease (Cappuccio et al., 2010; Sauvet et al., 2010). Moreover, SD promotes a proinflammatory and prothrombotic state with increased oxidative stress contributing to endothelial dysfunction (Tobaldini et al., 2017), also implied by our fNIRS measurements. Finally, at the cellular level, increased phagocytic activity of brain microglia and astrocytes has been revealed in an animal study (Bellesi et al., 2017) after SD that could be a putative mechanism for memory deficits. To sum up, these pathogenic factors of increased cardiovascular risk associated with short sleep duration (Tobaldini et al., 2019) could also be important in the long‐term cerebrovascular complications.

Concerning the limitations of this study, the small number of participants should be emphasized first, which is balanced by the homogeneity of the tested population and the within‐subject design similarly to other researches in the field (Verweij et al., 2014). We used fNIRS method that is able to capture cerebral hemodynamics, while a more direct assessment of neuronal activity (e.g., EEG) would have provided further insight into the effect of sleep deprivation on functional brain networks. In order to interpret changes in FC, it should also be kept in mind that fNIRS is capable of detecting changes in cortical activity only. Nevertheless, this noninvasive imaging technique enables the simultaneous assessment of local changes in the NVC which is a key physiological regulatory mechanism of cerebrovascular control. Our results demonstrate the sensitivity of global density to the combined effect of sleep deprivation and stimulus indicating changes in wiring cost. Conversely, graph theoretical parameters of functional integration or segregation do not provide further details about the underlying brain networks perhaps due to the relatively low number of channels. Although static assessment of FC neglects the time‐varying nature of brain networks, dynamic analysis of functional connectivity (Racz et al., 2018) was not reasonable in this case (but see a recent paper for such application [Li et al., 2020]), regarding the limited duration of examined brain states (10 s).

Continued effort in this field is necessary to elucidate the pathophysiological mechanism of sleep deprivation that could allow for identifying informative markers of altered cerebrovascular homeostasis and brain function. Insomnia and sleep deprivation are also prevalent in older adults (30%–48% are affected) (Patel et al., 2018). Importantly, the functional and vascular consequences of sleep loss bear an intriguing resemblance to the biological changes associated with aging (Csipo, Mukli, et al., 2019; Ferreira & Busatto, 2013; Harrison et al., 2000; Sala‐Llonch et al., 2015). It is likely that the age‐related impairment of neurovascular coupling (Mahalakshmi et al., 2020; Tarantini et al., 2017) (which is due to an age‐related impairment of endothelial function [Csiszar et al., 2019; Kiss et al., 2019; Lipecz et al., 2019; Tarantini, Valcarcel‐Ares, et al., 2019; Tarantini, Yabluchanskiy, et al., 2019; Wiedenhoeft et al., 2019]) may exacerbate the SD‐induced changes on functional connectivity. Future studies should investigate the synergistic effects of sleep deprivation and aging in details. Additional studies are also needed to determine whether sleep improving strategies and the restoration of circadian rhythm could reverse the negative impacts of SD on functional connectivity and cognitive functioning.

## 5 CONCLUSIONS

Our results demonstrated the impact of 24‐hr sleep deprivation on the global properties of frontal lobe networks when investigating resting‐state functional connectivity and its alteration in response to a finger‐tapping paradigm assessed by fNIRS. The interaction between sleep state and stimulus indicated a significantly reduced task‐related decrease in average number of functional connections after sleep deprivation. A significant relationship was found between these changes of functional connectivity and the cognitive performance changes detected by the executive function tests. While decreased neurovascular coupling responses in the prefrontal cortex provide a possible explanation for certain observations in this study, alterations of functional connectivity related to sleep deprivation could rather be ascribed to impaired transition of the examined brain networks during task performance. In order to better understand the mechanisms contributing to sleep deprivation‐induced changes in functional connections and their link to impaired cognitive performance in real‐life situations (e.g., decision making, driving), further longitudinal studies are needed. We propose that fNIRS‐based approaches are ideally suited for this task.

## ETHICS APPROVAL

The study protocol was approved by the Institutional Review Board of the University of Oklahoma Health Sciences Center and was conducted in compliance with the Helsinki Declaration.

## PATIENT CONSENT STATEMENT

Written informed consent was obtained from all subjects prior to participation.

## PERMISSION TO REPRODUCE MATERIAL FROM OTHER SOURCES

Not applicable.

## CLINICAL TRIAL REGISTRATION

Not applicable.

## CONFLICT OF INTEREST

The authors declare that they have no competing interests.

### PEER REVIEW

The peer review history for this article is available at https://publons.com/publon/10.1002/brb3.2135.

## Data Availability

In this study, we collected near‐infrared spectroscopy recordings and neuropsychological test scores. Deidentified data that support the findings of this study are available from the corresponding author [A.Y.].
